# A mathematical model of dysfunction of the thalamo-cortical loop in schizophrenia

**DOI:** 10.1186/1742-4682-11-45

**Published:** 2014-10-18

**Authors:** Nils Rosjat, Svitlana Popovych, Silvia Daun-Gruhn

**Affiliations:** Heisenberg Research Group of Computational Biology, Department of Animal Physiology, Institute of Zoology, University of Cologne, Zülpicher Str. 47b, 50674 Cologne, Germany

**Keywords:** Mathematical modeling, Phase oscillators, EEG, Synchronization

## Abstract

**Background:**

Recent experimental results suggest that impairment of auditory information processing in the thalamo-cortical loop is crucially related to schizophrenia. Large differences between schizophrenia patients and healthy controls were found in the cortical EEG signals.

**Methods:**

We derive a phenomenological mathematical model, based on coupled phase oscillators with continuously distributed frequencies to describe the neural activity of the thalamo-cortical loop. We examine the influence of the bidirectional coupling strengths between the thalamic and the cortical area with regard to the phase-locking effects observed in the experiments. We extend this approach to a model consisting of a thalamic area coupled to two cortical areas, each comprising a set of nonidentical phase oscillators. In the investigations of our model, we applied the Ott-Antonsen theory and the Pikovsky-Rosenblum reduction methods to the original system.

**Results:**

The results derived from our mathematical model satisfactorily reproduce the experimental data obtained by EEG measurements. Furthermore, they show that modifying the coupling strength from the thalamic region to a cortical region affects the duration of phase synchronization, while a change in the feedback to the thalamus affects the strength of synchronization in the cortex. In addition, our model provides an explanation in terms of nonlinear dynamics as to why brain waves desynchronize after a given phase reset.

**Conclusion:**

Our model can explain functional differences seen between EEG records of healthy subjects and schizophrenia patients on a system theoretic basis. Because of this and its predictive character, the model may be considered to pave the way towards an early and reliable clinical detection of schizophrenia that is dependent on the interconnections between the thalamic and cortical regions. In particular, the model parameter that describes the strength of this connection can be used for a diagnostic classification of schizophrenia patients.

## Introduction

Schizophrenia is a severe and complex mental illness causing disability [1–3]. It has been conceptualized as a disconnectivity syndrome concerning the interplay of the brain areas involved. As information on the activity of some of the deeply localized involved brain areas, such as the thalamus is not accessible to noninvasive electroencephalography (EEG) measurement, alternative methods, like mathematical models, need to be developed in order to deepen our understanding of the fundamental neural processes underlying schizophrenia, and to detect dysfunctions in the interactions between the participating brain areas. Such methods aim at deriving reliable criteria that indicate the progress of the disease at an early stage. The early recognition is considered to be of special importance in schizophrenia.

As schizophrenia has a very high degree of complexity, due to the large number of neuronal processes involved, there is no model that treats all aspects of the disease concurrently. The model in [4], for instance, focuses on the empirical dopamine hypothesis of schizophrenia and postulates that an imbalance between glutamate and dopamine activity plays a key role in schizophrenia disorder. In particular, the authors could show that both hypoglutamatergia and hyperdopaminergia result in reduced activation of the striatal complex and thus leads to schizophrenia [4].

Heiden et al. [5] on the other hand modeled the basic neural circuit underlying schizophrenia as a dynamical system on a microstructural level of pyramidal cells (see also Mackey et al. [6]). In their model, periodic firing patterns were associated with healthy behavior, whereas aperiodic/chaotic firing patterns were associated with schizophrenic states and the switch from periodic to aperiodic firing took place due to an increase in dopamine level. In [7], the same model was analyzed in a hypo-glutamatergic setting, and it also exhibited aperiodic firing.

A top-down approach in modeling the symptoms of schizophrenia is proposed in [8, 9]. These authors relate cognitive, negative and positive symptoms of schizophrenia to a reduced depth of attractor basins of the model regarded as a dynamical system. The model consisting of pools of leaky integrate-and-fire neurons for the involved pyramidal cells and inhibitory interneurons and AMPA, NMDA and GABA _A_ synapses either developed spontaneous firing or was attracted to a high-firing state. The decrease in NMDA-receptor conductance led to a decrease in attractor stability and therefore a decrease in memory and an increase in distractability [8, 9]. An additional decrease in the conductance of GABA-synapses led to jumping from spontaneous activity to attractors which could be identified with the increase of positive symptoms [8].

In [10], fMRI data of healthy and schizophrenic test persons obtained in a memory-task experiment were used to assess the connectivity between the visual, parietal and prefrontal regions using DCM as proposed by [11]. Each of these regions has a self-coupling and bidirectional all-to-all coupling. Moreover, the working memory modulates the coupling from visual to prefrontal and from prefrontal to parietal regions. The schizophrenic patients and the healthy subjects differed significantly in the self-coupling strengths, in the coupling between parietal and prefrontal regions in both directions and in the coupling from visual to prefrontral regions [10].

In contrast to the modeling studies described above, we focused our attention on the phenomena observed on the large-scale level of dysfunction of the thalamo-cortical loop in schizophrenia. We did therefore not include any explicit biophysical properties into our model. We constructed a mathematical model based on a study by [12]. In this study, the difference between healthy subjects and schizophrenia patients was investigated, using individual EEG recordings with respect to phase locking (PL) in the four frequency bands (*θ*, *α*, *β* and *γ*). The two groups of test persons showed significant difference in the strength of PL in the *θ*- and *α*-frequency band, while no significant difference in PL was observed in the other two frequency bands. The duration of PL, i.e. the time until the system desynchronize again, differed for each frequency band. However it did not differ significantly between healthy subjects and schizophrenia patients in each of the four frequency bands.

In our earlier model [13], we described each component of the thalamo-cortical loop as a single phase oscillator, where each of them operated at its natural frequency. We used phase oscillators for the description of the dynamics in each brain area, since previous studies suggested that the timing of the brain rhythms, i.e. the phases, were more important than their amplitudes [12]. The coupling between these oscillators was expressed in form of weighted phase differences with suitable coefficients, which were determined from the natural frequencies of the oscillators. Using this model, we were able to explain the difference in phase locking in the respective frequency bands of the two groups of test persons depending on the feedback from the cortex to the thalamus. In this model, however, the synchronization effect observed directly after the given auditory stimulus did not abate and vanish after a given period of time, contrary to what was seen in the experiments [12]. We had therefore to change our mathematical model in order to be able to account for the desynchronization effects, too.

In the present study we extended this mathematical model such that now each area of the thalamo-cortical loop is represented by a large population of phase oscillators. The coupling between populations is driven by a complex meanfield (definition see below). To reduce this high-dimensional model to a low dimensional system which still reflects the behavior observed in the EEG data and to allow its analysis, we use the reduction methods of Watanabe- Strogatz [14], Ott-Antonsen [15] and Pikovsky-Rosenblum [16]. The mathematical analysis of the model offers a conducive explanation for the underlying mechanisms leading to the differences observed between healthy subjects and schizophrenia patients, as seen in the experiments by [12]. Our results suggest that the differences are due to a decrease in strength of the coupling from the auditory cortex to the thalamus in schizophrenia patients. Even so, our model is a rather abstract description of the neural dynamics that take place in the thalamo-cortical loop. A decrease in coupling strength can occur due to changes in the dopamine, glutamate or serotonin concentrations. This means in any case a reduction in signal transduction from the auditory cortex to the thalamus. Furthermore, our analysis of the reduced system reveals that the mechanism underlying the abolition of synchrony observed in all four brain wave bands is based on a fold limit cycle bifurcation that takes place when the coupling between the auditory cortex and the thalamus is changed (in either direction). Our model additionally predicts that a change in coupling strength from the thalamus to the auditory cortex, however, affects the duration of phase synchrony.

The paper is organized as follows. In section “The experimental setup and results”, we review the experimental setup and the results of the study by [12]. In section “Mathematical model”, we present the general structure of the thalamo-cortical loop and set up a mathematical model which we use to analyze first the behavior of two coupled brain regions, the thalamus and one cortical region, and then that of three coupled ones, the thalamus and two cortical regions.

## The experimental setup and results

**Figure 1:**
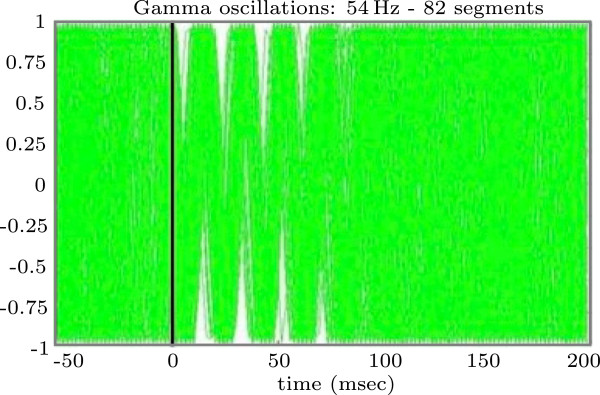
**Experimental data.** The cosine of the phases, derived from EEG data of a single participant, expressing a frequency of 54 Hz is shown (from [13]).

The mathematical model presented below is based on experimental results (for details of the experiment and methods used see [12]). In the following, we outline the experiment performed in [12]. Two groups of participants were investigated during the experiment: the first group consisted of 32 schizophrenia patients and the second of 32 healthy subjects. The experiment was based on the well-established paired click paradigm [17]. It consisted of 96 paired clicks (*S*_1_ and *S*_2_). Each click had a duration of 1 ms. The interstimulus interval between the two clicks within a pair lasted 500 ms, and the inter-trial interval between pairs of clicks 10 s. The EEG was continuously recorded using 32 electrodes during the whole experiment. Data from the vertex electrode Cz were taken for the analysis, because the cortex around the location of this electrode performs sensory and motor functions, see [18]. The recorded data have been divided into epochs of 1500 ms (500 ms prior to *S*_1_ and 500 ms following stimulus *S*_2_). The occurence of stimulus *S*_1_ in each segment was set to *t*=0, hence the stimulus of each segment appeared at *t*=0. To obtain detailed information on the temporal and spectral properties of the EEG, a single-trial analysis was applied to the epochs. Thus a complex Morlet wavelet transformation in the frequency range from 3 Hz to 60 Hz in 1 Hz steps was performed to compute the phases of the single-trial data. A typical result is displayed in Figure 1 (adapted from [13]) where the cosine of the single trial phase after the wavelet transformation for a fixed frequency 54 Hz is shown. It includes 82 superimposed segments. Uniform distribution of the phases prior stimulus onset, i.e. for *t*∈[ -50,0] is clearly visible, while the so called phase locking effect after the stimulus, i.e. for *t*∈[ 0,75], and the effect of desynchronisation after *t*=75 ms can also be clearly discerned.

The stimulus locking index
1

defined in [19], can be used to measure the degree of phase locking for a certain frequency at time t. We denote the number of repetitions of the auditory double clicks by n and the phase of the k-th oscillation at time t by *ϕ*_*k*_(*t*). Values near 0 indicate a uniform distribution of phases and values near 1 nearly synchronized phases. It was found that schizophrenia patients produced significantly less phase locking in lower frequency bands after the first stimulus than healthy subjects [12].

Table 1 shows the *SLI* and the duration of synchronization for the two groups of subjects for the *θ*- and *α*-frequency band for which significant differences were found in the *SLI* (see [12]). Based on these experimental results, we constructed a mathematical model of the thalamo-cortical loop and used it to explain the observed differences between the neural activities of schizophrenia patients and healthy subjects.

**Table 1:** **Example behavior of cortical regions**

	Max. SLI		Duration
	Patients	Control	[ms]
*θ*	.30	.37	400
*α*	.19	.26	250

Columns 1-2: Maximum SLI values (from [12]); Column 3: Approximate duration of stimulus responses ([12], Figure two).

## Mathematical model

**Figure 2:**
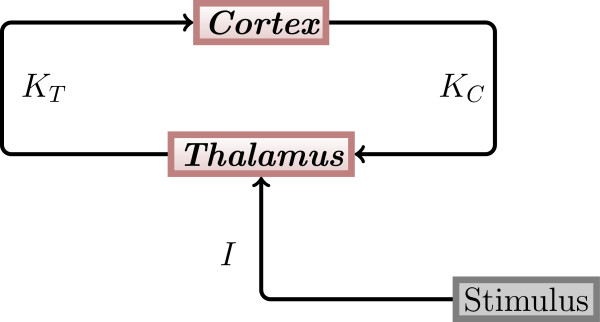
**Thalamo-cortical loop.** The general structure of the thalamo-cortical loop used in our modeling study. *K*_*T*_ and *K*_*C*_ denote coupling strengths between the thalamus and the auditory cortex. Both the thalamus and the cortex are represented by one population of oscillators each. I denotes the stimulation strength.

Our model of the thalamo-cortical loop is based on the results of [4, 12, 13, 20]. According to the experimental findings in these sources, we assume that essentially three main brain areas are actively involved in auditory signal processing, i.e. the thalamic auditory relay nucleus (here for the sake of simplicity, they are referred to as thalamus), the thalamic reticular nucleus (here named TRN) and areas of the auditory cortex. An auditory input signal reaches the thalamus and then propagates to the auditory cortex. From the auditory cortex, the signal propagates to higher cerebral regions such as the prefrontal cortex and back to the TRN, which inhibits the thalamus. Furthermore, backpropagation from higher regions such as the prefrontal cortex modulates the activity of the thalamus. These inhibitory and modulating influences lead to a reduced response of the thalamus to the second of the two clicks [21–23]. Since we are only interested in the dynamics after the first and before the second stimulus, we neglected the impact of the TRN. This means that only the thalamus and different regions of the auditory cortex are present in our model. It can be assumed that the different cortical regions act in different oscillatory frequency ranges, which correspond to the *θ*, *α*, *β* and *γ* ranges. The structure of the thalamo-cortical loop used in our model is shown in Figure 2.

In our earlier work [13], we described each part of the thalamo-cortical loop by a single phase oscillator. Each oscillator had a natural frequency, which was chosen according to biological/experimental observations. We assumed, that coupling between all phases is a form of weighted phase difference with suitable coefficients to be determined from the natural frequencies. With this model, it was possible to reproduce the effects that correspond to phase locking as observed in the EEG data of the two groups of subjects. However, in contrast to what the data showed (see Figure 1, t >75 ms), the oscillators remained in the synchronized state perpetually, and their phases did not desynchronize again. To overcome this major drawback of our old model, we now describe each element of the thalamo-cortical loop as a large ensemble of nonidentical phase oscillators. Each oscillator in the population has a natural frequency *ω*, which is chosen from a Lorentz distribution *n*(*x*). The coupling between populations is driven by a complex meanfield. In the experiments, repeated stimulations were used in order to obtain stimulation moments at different phases. In our mathematical model we use 1000 oscillators with distributed phases in each population and stimulate each oscillator at *t*=0. Since we choose the initial conditions for each oscillator to be different, the results obtained by stimulating them at only one point in time are comparable to the experimental conditions.

First, we will consider a minimal mathematical model, which consists of only two populations of oscillators, one for the thalamus and one for the *θ*-frequency band of the auditory cortex. We will use this simplified model to understand the mechanism behind the transition from the synchronized to the desynchronized state after stimulation (as seen in Figure 1).

### Minimal mathematical model (two populations)

**Figure 3:**
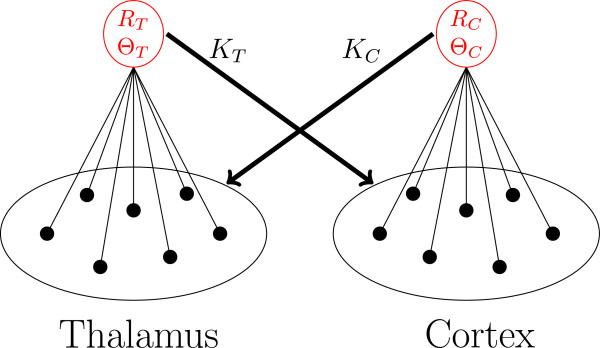
**Mean field coupling.** Visualization of the complex mean field coupling between the thalamic region (*T*) and a single cortical region (*C*). Black represents the individual oscillators and red their complex mean fields. The oscillators of one population are coupled through the mean phases *Θ* and the degree of synchronization *R* of the oscillators in the other population. See text for details.

In the minimal model, two populations of oscillators are coupled via their complex mean fields as shown in Figure 3. One of them describes brain wave activity in the thalamus and the other one in the cortex, in this case in the *θ*-band. In the course of this work, we will refer to these populations as thalamus population and cortex population, respectively.

The system describing the neural activities of the two populations reads as follows
23

where *ω*_*T*_ and *ω*_*C*_ are continuous parameters distributed in each ensemble of oscillators as
4

and represent the natural frequencies of the oscillators. *ϕ*_*T*_ and *ϕ*_*C*_ denote the phases of the oscillators of the thalamus and cortex populations, respectively. The state of each population can be described by the distribution density *W*(*x*,*ϕ*,*t*)=*n*(*x*)*w*(*x*,*ϕ*,*t*), with the conditional distribution density of oscillators denoted by *w*(*x*,*ϕ*,*t*) [16].

Each oscillator in the cortical population is coupled to the complex mean field
5

of the thalamic population and each oscillator in the thalamic population is coupled to the complex mean field
6

of the cortical populations (see Figure 3). Coupling strengths are denoted by *K*_*T*_ and *K*_*C*_, respectively.

The thalamus population is stimulated by an external stimulus that acts directly on it (see Figure 2). This stimulus is represented by the term *I*(*t*) cos(*ϕ*_*T*_) where
7

A complete analysis of the model can be performed by means of the Watanabe-Strogatz (WS) theory [14]. With this theory an N-dimensional system of identical oscillators can be reduced to a three-dimensional system with the global variables *ρ*, *ϕ* and *ψ*. Here *ρ* is the global amplitute, *ϕ* and *ψ* are global phases. The original phase variables can be reconstructed from the obtained WS variables by means of the time-dependent transformation. The theory is described in more detail in the Appendix.

Following [14–16, 24], we transform system (2)-(3) using Equation 36 (see Appendix) and obtain a reduced system of WS equations with the new variable *ρ*_*T*_, *ϕ*_*T*_, *ψ*_*T*_ and *ρ*_*C*_, *ϕ*_*C*_, *ψ*_*C*_. By additionally introducing  and the phase shift *α*_*a*_(*ω*_*a*_)=*ϕ*_*a*_-*ψ*_*a*_, (*a*=*T*,*C*), we obtain
891011

Here,  and *A*^∗^ denotes the conjugate complex of *A*.

Now we consider this reduced set of equations with respect to the Ott-Antonsen manifold [25]. In this case, *z*(*ω*) no longer depends on *α*(*ω*), and the mean fields *Y*_*C*_ and *Y*_*T*_ can therefore be written as
12

Following the work by Ott and Antonsen for a similar distribution [25], the integrals in Equation (12) can be calculated by applying the residue theorem, under an additional assumption that *z*_*a*_(*ω*) (*a*=*T*,*C*) is analytic in the upper half-plane. This calculation yields
1314

Thus Equation (8) for  and Equation (10) for  provide a 2-dimensional system of complex ODEs that describe the behavior of the order parameter of the thalamic and the cortex population, respectively:
1516

In the following, we will investigate this system of two complex differential equations, i.e. its dynamics during the post-stimulus interval.

#### Analysis of the model behavior in the post-stimulation interval

For the analysis of eqs. (15)-(16) in the post-stimulus interval, i.e. when *I*(*t*)=0, we transform them to a 4-dimensional system of real ODEs via *Y*_*T*_=*x*_*T*_+*iy*_*T*_ and *Y*_*C*_=*x*_*C*_+*iy*_*C*_. This leads to
17181920

In a next step, we linearize this new system about its fixed point *x*_*F*_=(0,0,0,0) and investigate the stability of this fixed point with the coupling strengths *K*_*C*_ and *K*_*T*_ as parameters. The linearized system reads:
2122

**Figure 4:**
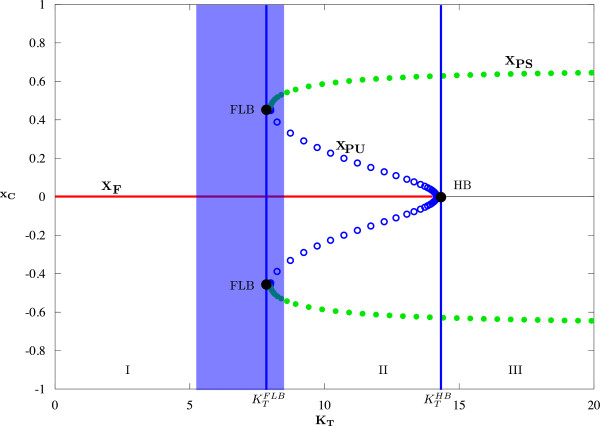
**Bifurcation diagram.** Bifurcation diagram in the (*K*_*T*_-*x*_*C*_)-plane. The coupling strength from the cortex to the thalamus has been set to *K*_*C*_=1.2 and the coupling strength *K*_*T*_ from the thalamus to the cortex is varied;  Hz and  Hz. Red line: stable fixed points (*x*_*F*_), black line: unstable fixed points; green filled circles represent the maximum and minimum of stable periodic orbits (*x*_*PS*_) while the blue circles represent the maximum and minimum of unstable periodic orbits (*x*_*PU*_). Bifurcation points, i.e. the fold limit cycle bifurcation (FLB) at  and the Hopf-bifurcation (HB) at  are marked with vertical blue lines. The light blue box represents the parameter region where the initially synchronized system desynchronizes in finite time (like in Figure 1).

A one-dimensional bifurcation diagram is displayed in Figure 4. For the calculation of this diagram, we fix one of the coupling parameters, *K*_*C*_=1.2, and show the dependence of one of the system variables (*x*_*C*_) on the second coupling parameter *K*_*T*_. The system has a fixed point *x*_*F*_=(0,0,0,0), which is stable for . At  a Hopf bifurcation (HB) occurs in the system, i.e. a complex conjugate pair of eigenvalues of A passes through the imaginary axis (see Figure 4). At this point, the branch of stable fixed points (*x*_*F*_, red line in Figure 4) loses its stability because it collapses into a branch of unstable periodic orbits (*x*_*PU*_, blue circles). Additionally, the system exibits a fold limit cycle bifurcation (FLB) at . At this bifurcation point, two periodic orbits, a stable (*x*_*PS*_, green discs) and an unstable one (blue circles) are born. The bifurcation diagram reveals three parameter regions in which the system displays different behavior. In region I  we have a stable fixed point *x*_*F*_=(0,0,0,0), which corresponds to the state of full desynchronization in the non-reduced system (2)-(3). In region III  the fixed point *x*_*F*_ has lost its stability and all trajectories are attracted to the stable periodic orbit *x*_*PS*_ (filled green circles in Figure 4). This corresponds to a state near perfect synchronization of the non-reduced system. In region II , the system is bistable. It can exhibit fixed point solutions as well as periodic ones. Both behaviors are separated by an unstable periodic orbit *x*_*PU*_. Depending on the initial conditions of the system, the trajectory will stay in the region of attraction of the fixed point *x*_*F*_ or is attracted by the stable periodic orbit *x*_*PS*_.

Let us now focus on the blue region surrounding the bifurcation points FLB. It is possible to choose a value of the parameter *K*_*T*_ inside this region such that the trajectory is resetted to a state near the periodic orbit and drops back to the stable fixed point after a certain amount of time.

**Figure 5:**
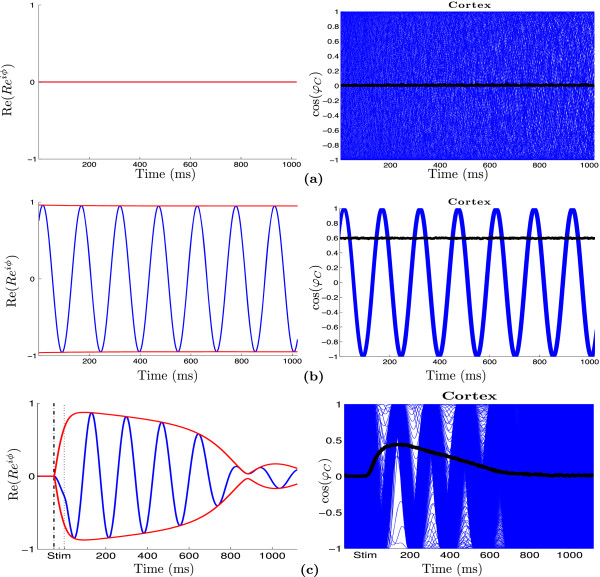
**Bifurcation behaviors.** The real part of the solution of the reduced system (left) and the corresponding cosine of the solution of the non-reduced system (right) for **(a)** *K*_*T*_=1 in region I, **(b)** *K*_*T*_=16 in region III and **(c)** *K*_*T*_=5.5 in region II of Figure 4 are shown. In (c) the behavior of both systems is shown during and after stimulation. For the reduced system, red lines indicate the maximum amplitude of the solutions (panels in the left column). For the non-reduced system, the oscillators’ (only 200 out of *N*=1000 displayed) activities are shown as blue curves, and the corresponding max SLI is plotted in black (panels in the right column). *K*_*C*_=1.2,  Hz,  Hz. The stimulus intensity was set to *I*=100 and had a duration of 50 ms for all simuli.

Figure 5 shows the behavior of the reduced system (left) and the corresponding behavior of the non-reduced system (right) for the three parameter regions described above. Red lines in the figures of the left column indicate the maximum amplitude of the solutions. Black thick lines in the figures of the right column indicate the SLIs. In region I (see Figure 4), i.e. for low value of *K*_*T*_, e.g. *K*_*T*_=1, the reduced system exhibits a stable fix point solution (Figure 5(a), left), and the corresponding non-reduced system is in the desynchronization regime (Figure 5(a), right), hence SLI = 0. For *K*_*T*_ in region III (see Figure 4), e.g. *K*_*T*_=16, the stable periodic orbit of the reduced system is shown in Figure 5(b) on the left and the corresponding synchronization regime of the non-reduced system on the right with SLI = 0.6. In Figure 5(c) *K*_*T*_ is fixed near  (*K*_*T*_=5.5, left edge of blue region in Figure 4). With this parameter choice, we observe the same dynamics as seen in the experiments: before stimulation we have desynchronization in the non-reduced system and a fix point in the reduced one. After the stimulation interval (marked with vertical dash lines), we see a phase reset, and the phases of the oscillators of the non-reduced system are now synchronized (thin wave). After some time (t >700 ms) they desynchronize again. The length of the synchronization state can be modulated by changing the distance of *K*_*T*_ to . The closer *K*_*T*_ is set to  the longer the trajectory will stay in the state of synchronization before it desynchronizes again.

**Figure 6:**
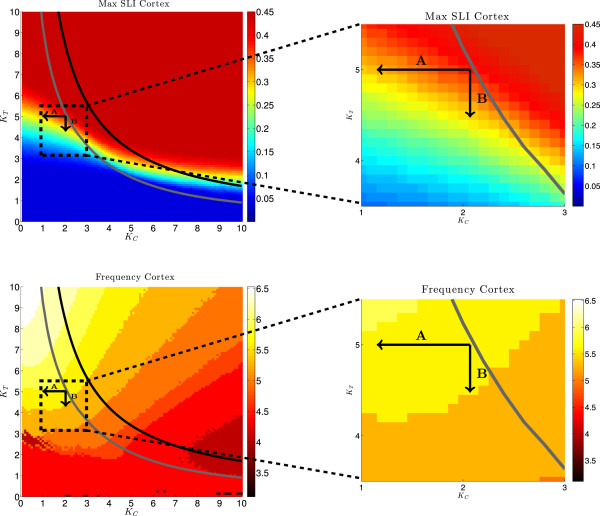
**Coupling parameter dependence.** Left: Maximal SLI (top) and mean frequency (bottom) of the oscillators in the cortical region (for  Hz,  Hz and *N*=1000) as functions of the coupling strengths *K*_*C*_ and *K*_*T*_. Right: enlargments of the marked areas in the corresponding panels. Solid curves mark Hopf-Bifurcations (black) and fold-limit cycle bifurcations (gray) of the reduced system. The arrows A and B show possible transitions from a level of high synchronization (*K*_*C*_=2,*K*_*T*_=5) to a level of lower synchronization (tip of arrow A: *K*_*C*_=1.1,*K*_*T*_=5; tip of arrow B: *K*_*C*_=2,*K*_*T*_=4.5).

In Figure 6, top left, the maximum SLI of the cortex population for different pairs of coupling strengths (*K*_*C*_,*K*_*T*_) is shown. For this we stimulated the system of two populations (*N*=1000 oscillators each) with a stimulation strength of *I*=100 for 50 ms and calculated  for each fixed pair (*K*_*C*_,*K*_*T*_). The dependence of the peak of the frequency distribution on these parameters was calculated using the fast Fourier transformation (MATLAB function FFT). It is shown in the bottom left panel of Figure 6. The coupling strengths *K*_*T*_ and *K*_*C*_ were varied independently from 0 to 10 with a step size of 0.05. The grey and black curves in the *K*_*C*_- *K*_*T*_ plane represent the branches of fold limit cycle bifurcations (FLBs) of the periodic orbits and of Hopf-bifurcations (HBs) of the fixed points, respectively. As seen in Figure 4, only parameter values to the left and close to the grey curve and parameter values which yield *SLI*≥0.3 guarantee a strong synchronized population which desynchronizes in finite time. The closer the coupling parameters are set to the branch of FLBs the longer the population will stay in the synchronized state. When the value of the coupling parameter *K*_*T*_ is decreased the system moves away from the curve of FLBs, thus the synchronization of the system becomes weaker and shorter. This happens even for small changes in *K*_*T*_ (direction denoted by B in Figure 6, top left). Changing the value of the coupling parameter *K*_*C*_, however, has a much weaker effect on the strength of synchronization (direction denoted by A in Figure 6, top left). An enlargement of this region of interest is shown in Figure 6, top right.

We know from the experimental data of [12] that schizophrenia patients show a lower synchronization in the *θ*-band than healthy subjects (max SLI = 0.3 and 0.37, respectively). If we now change the parameters *K*_*C*_ and *K*_*T*_ such that the SLI changes from 0.37 to 0.3 (in the A or B direction or in a direction representing a linear combination of the two), the mean frequency of the *θ*-oscillators does only minimally change, i.e. it remains approximately 6 Hz. A major change in the mean frequency should of course not happen when the conditions of the system are changed from the healthy to the schizophrenic state. Figure 6, bottom left shows the mean frequency of the cortical oscillators for different pairs of coupling parameters (*K*_*C*_, *K*_*T*_). An enlargement of the region of interest is shown in Figure 6, bottom right.

Summing up, our model simulations nicely show that the neural dynamics observed in EEG data of schizophrenia patients and healthy subjects strongly depend on the strength of the coupling between the thalamus and the cortex: decreasing one or both of the coupling parameters *K*_*T*_ or *K*_*C*_ in an appropriate manner decreases the max SLI of the system and changes the time the system stays synchronized but leaves its mean frequency nearly unchanged.

### Model with three populations

In this section, we consider the extension of our minimal model to three populations of coupled phase oscillators, where one of them describes brain activity in the thalamus and the other two *θ*- and *α*-brain waves in the auditory cortex. We only consider the *θ*- and *α*-frequency band here since the experimental data of [12] showed significant differences between schizophrenia patients and healthy subjects in these frequency bands, only.

Our extended system has the form
232425

All notations are the same as in the minimal model. *ϕ*_*T*_ and ,  are the phases corresponding to the oscillators of the thalamus population and the oscillators of the *θ* cortex and *α* cortex populations, respectively. *ω*_*T*_,  and  represent the natural frequencies of the corresponding oscillators chosen from the distribution described in Equation 4.

**Figure 7:**
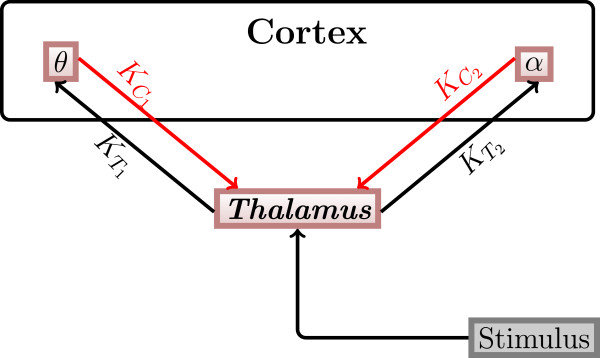
**Three population structure.** Scheme of the three-population model. Fixed couplings are shown in black and variable ones in red.

Each oscillator of both cortical populations is coupled to the complex mean field


of the thalamic population with the corresponding coupling parameters , *j*=1,2. Each oscillator in the thalamic population is coupled to both complex mean fields


of the *θ* and *α* cortical populations with the corresponding coupling parameter , *j*=1,2.For the sake of simplicity and as a first approximation, we assume, that there is no direct connection between the two cortical populations. Note, that these populations are considered as functionally distinct groups in the cortex, not as anatomically distinct ones. They, however, influence each other indirectly through the feedback they receive from the thalamic population. Figure 7 shows a schematic illustration of the thalamo-cortical loop with two cortex populations. The thalamus population is stimulated with the same external stimulus as in the case of the minimal model (see Equation 7).

Again, we apply the Watanabe-Strogatz ansatz [14] and its extension by Pikovsky and Rosenblum [16] to the system (23)-(25) and obtain
262728293031

This system can again be reduced to a 3-dimensional system of complex ODEs representing the dynamics of the order parameters. The reduced system has the form:
323334

**Figure 8:**
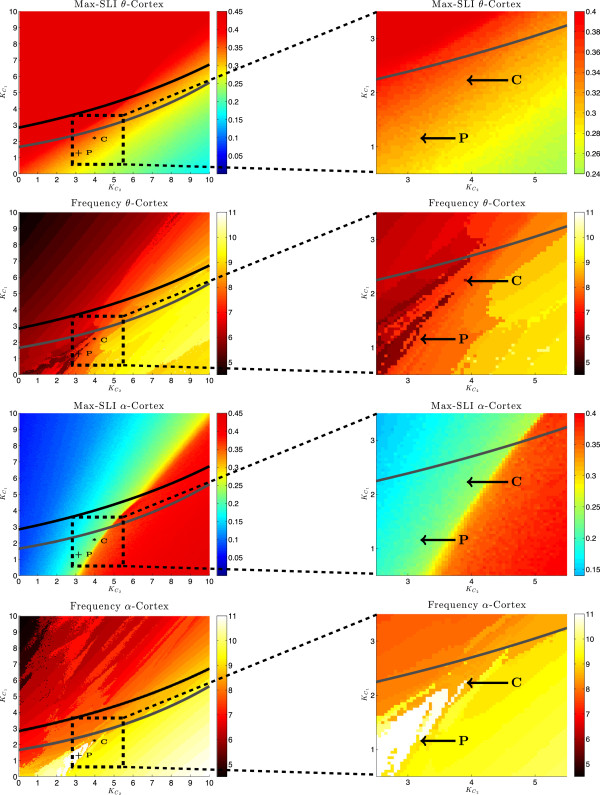
**Coupling parameter dependence for three populations.** Left: maximum SLI and frequency of the *θ*-population ( Hz; top two) and the *α*-population ( Hz; bottom two) as functions of the coupling parameters  and . Right: enlargement of the marked areas. The points in the corresponding left panels C () and P () mark parameter choices that yield the max SLIs and frequencies observed in healthy subjects (controls) and schizophrenia patients, repectively. The coupling parameters from the thalamus ( Hz) to the cortex are fixed at  and . Solid curves mark branches of Hopf bifurcations (black) and of fold limit cycle bifurcations (gray) of the reduced system.

The main results of the analysis of this model are illustrated in Figure 8. The panels of the figure on the left show the maximum SLI and the frequency for both cortex populations over the plane of the coupling parameters  and . The right column shows enlargements of a region of interest of each of the figures shown in the left column. In these simulations we fixed the coupling from the thalamus population to the *θ*- and *α*-populations, i.e.  and , respectively. For each parameter pair , we stimulated the system (*N*=1000 oscillators in each of the three populations) with a stimulus intensity of *I*=100 for 50 ms and calculated the maximum SLI as . The results are shown in the first and third row of Figure 8. The figures in the second and fourth row of Figure 8 show the mean frequency of the *θ*- and *α*-populations, respectively, as functions of the coupling strengths  and . The coupling strengths  and  were varied independently from 0 to 10 with a step size of 0.05. The grey and black curves again denote the branches of fold limit cycle bifurcations (FLBs) of the periodic orbits and of Hopf bifurcations (HBs) of the fixed points, respectively.

Using these simulations, we can now navigate through the  parameter plane and find pairs of coupling parameter values at which our model exhibits brain dynamics as observed in healthy subjects or in schizophrenia patients. To change the dynamics of the model from the healthy to the schizophrenic state, the parameter pairs need to be chosen such that: i) the max SLI of the *θ*-brain waves changes from 0.37 to 0.3; ii) the one of the *α*-waves from 0.26 to 0.19; iii) the frequencies of the *θ*- and *α*-populations change only minimally, i.e. they remain approximately 5-7 Hz for the *θ*- and 9-12 Hz for the *α*-population.

With the letters *P* and *C*, we label the positions in the  parameter plane which correspond to the max SLIs and frequencies observed in schizophrenia patients and healthy subjects, respectively. For healthy subjects the coupling constants are , for schizophrenia patients . We can see that schizophrenia patients, compared to healthy subjects, have a reduced feedback from both cortex populations to the thalamus.

We therefore hypothesize based on our model that schizophrenia patients have deficits in signal transduction from the auditory cortex back to the thalamus.

**Figure 9:**
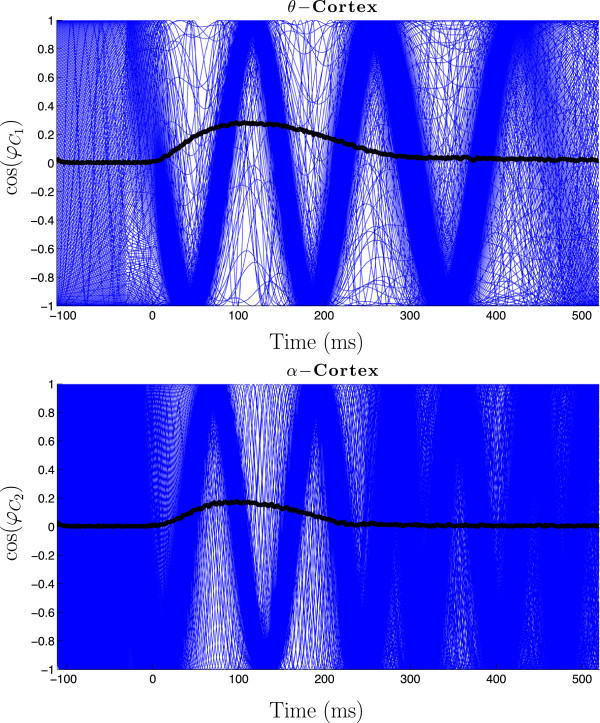
**Dynamic behavior of cortical regions.** An example of the desynchronization in the two cortical areas of the non-reduced system (only 200 of *N*=1000 oscillators are shown);  Hz  Hz,  Hz, , , , . The max SLI is shown in black. Frequency of coupled oscillators in the phase-locking interval: 6 Hz for *θ*-population and 9.4 Hz for *α*-population.

Figure 9 shows the behaviour of the *θ*- and *α*-populations in the case of schizophrenia patients, i.e. the coupling parameters are , , . The cosine of the phases of 200 oscillators is shown (in total we calculated N=1000 oscillators in each population). The system was stimulated at *t*=0. The black lines indicate the max SLI. Before the stimulation, i.e. *t*∈[ -100,0], we have a uniform distribution of the phases, which means that the oscillators in each population are desynchronized, i.e. max SLI = 0. Directly after the stimulation at *t*=0 a phase reset occurs and the phases of the oscillators synchronize (thin blue waves), hence now max SLI >0. After a certain time, they desynchronize again: the desynchronization in the *α*-population happens earlier (at ≈250 ms) followed by that in the *θ*-population (at ≈400 ms). These simulation results fully agree with the experimental data [12].

## Discussion

We have constructed a mathematical model which describes some aspects of the dysfunction in neural activity of the thalamo-cortical loop during schizophrenia. This model is, in contrast to the models introduced at the beginning [5, 7, 8, 10], an abstract description of the neural activity of the brain regions involved. The description is based on synchronization phenomena as found in the EEG records of healthy subjects and schizophrenia patients. The model allows us to study the interaction of the brain areas involved in a systematic way and to detect the effects of changes of the couplings between them.

A major advantage of our model is that it provides insight into activities of brain areas not directly accessible by EEG measurements. A second advantage is its flexibility: since we did not include any explicit biophysical properties into our model and stayed on a phenomenological macrostructural level, it can be adjusted to fit similar effects in other diseases, e.g. Morbus Parkinson [5] or bipolar disorder [26]. Patients suffering from Morbus Parkinson show a decrease in post-movement synchronization in the *β*-band [27] in the motor cortex. To simulate the system dynamics underlying this disease, it is possible to extend our model by including oscillators with *β*-frequencies in the cortex population (as done by the extension of our model from initially 2 to 3 populations). In a graph-theoretical analysis, Kim et al. [26] could show that only synchronization in *α* frequencies is significantly lower in patients with bipolar disorder than in healthy controls. Thus it would be possible to adjust the coupling strengths in our model in a way that it could appropriately reproduce the symptoms of this disease.

We have seen that the description of each area by a population of phase oscillators with distributed frequencies instead of only a single one per region allows the model to exhibit phase desynchronization in addition to phase-resetting after a given auditory stimulus. With this model it is now possible to get insights into the brain dynamics of the thalamic region which are not accessible to EEG measurements and to investigate the impact of different connection topologies between the thalamic and cortical brain regions on the duration and strength of synchronization in the respective brain frequency bands.

By analyzing our model, we have seen that it supports the current view [9] that the coupling between the thalamic and the cortical regions is responsible for dysfunction of the thalamo-cortical loop in schizophrenia. In particular, our model shows that a reduction in the strength of coupling from the thalamic to a cortical region, i.e. decreasing the coupling parameters , shortens the time interval of phase synchronization after stimulus. The closer  is to the fold limit cycle bifurcation the longer the state of high synchrony lasts. A reduction in the strength of the coupling from the cortical regions back to the thalamus, i.e. decreasing the coupling parameters , however reduces the strength of synchronization. Comparing this with the findings in [12] yields that patients differ mostly in the *θ*- and *α*-frequency bands in that they have a significantly lower coupling  from the cortex to the thalamus and only a little weaker coupling  from the thalamus to the cortex, than healthy subjects.

The bifurcation analysis of our model was only possible, because we were able to reduce this large-scale model to systems of only two or three dimensions by using the Ott-Antonsen theory and the reduction methods by Pikovsky and Rosenblum. Thanks to these reduction methods, we could do so without loosing the desynchronization phenomena observed in experiments and exhibited by the large-scale model. The bifurcation analysis helped us to understand the mechanisms underlying the desynchronization which follows the initial phase reset after the auditory stimulus. As far as we are aware of, our model can show for the first time which bifurcations underlie the changes in the simulated brain dynamics.We have, furthermore, seen that the mechanism of desynchronization is preserved if the model is extended from initially 2 to 3 regions to describe the neural activity in the thalamo-cortical loop. In this model, however, both parameter regions which correspond to data of schizophrenia patients and of healthy subjects are rather small (see little island in Figure 8). For the sake of simplicity and as a first approximation, we did not include any direct couplings between the cortical regions. Preliminary results (simulation results not shown) suggest that the size of this parameter region will increase, if additional intra-cortical connections are included in the model, and the mechanisms of desynchronization will still be preserved. However, further investigations will be needed to gather firm evidence that supports the preliminary results.

Simulation results obtained with our model support the notion that schizophrenia is not caused by focal brain abnormalities, but results from pathological interactions between brain regions [28]. In contrast to other studies that found abnormal functional connectivity between temporal and frontal regions, as measured by PET and fMRI [29–31], our model hypothesizes that it is the feedback from the thalamus to the auditory cortex that causes the disabilities, even in the absense of direct coupling between cortical frequencies.

Of course, the validity of our model needs to be further tested by comparing the simulation results with experimental ones obtained in new measurements. The “disconnection hypothesis” suggests that the core pathology of schizophrenia is an impaired neuromodulation of synaptic plasticity that leads to abnormal functional integration of neural systems, i.e., “dysconnectivity” [32, 33]. A possible next step would therefore be to investigate the influence of medications like eicosapentaenoic acid [34], pregnenolone [35, 36] or antipsychotics [37] on the functional signal transduction by calculating individual SLIs from the EEG data or in a DCM study [38]. This would provide information on the coupling between the thalamus and the auditory cortex in schizophrenia patients. One could then compare whether a regeneration of functions can be linked to a restored connection strength between thalamus and cortex.

Moreover, EEG measurements on individual patients and the calculation of their individual coupling strength between the thalamus and the cortex could then be used as a tool to diagnostically classify the different types of schizophrenia. The advantage of this approach would be that the grouping of patients would not simply be driven by data but would be constrained by a well-founded and carefully specified theory.

In order to explain the experimental observations we consider in this paper a simple and plausible model which inherits properties of the coupling of the Kuramoto model. Despite the simplicity of the model, we observed good agreement between experimental data and numerical simulations. This justifies our chosen degree of approximation. Of course more sophisticated phase models including the nonresonance case [39, 40] as well as detailed networks of the brain regions of interest could improve the quality of that results. Particularly, if we extended our model to include other brain regions with distinct natural frequencies, such as *β*- or *γ*-bands, we should definitely consider models that incorporate the nonresonance property.

## Conclusion

Our model can explain functional differences seen between EEG records of healthy subjects and schizophrenia patients on a system theoretic basis. Because of this and its predictive character, the model may be considered to pave the way towards an early and reliable clinical detection of schizophrenia that is dependent on the interconnections between the thalamic and cortical regions. In particular, the model parameter that describes the strength of this connection can be used for a diagnostic classification of schizophrenia patients.

## Appendix

### Mathematical theory

In this section, we will present the main reduction methods that are used to analyze our systems in section “Mathematical model”. The Pikovsky-Rosenblum ansatz [16] is an extension of the reduction method by Watanabe and Strogatz [14] which covers infinitely large systems of nonidentical phase oscillators. The general model treated in this theory reads:
35

The natural frequencies of the oscillators are denoted by *ω*(*x*,*t*). They depend on a continuous parameter x. The oscillators are coupled via a complex field *H*(*x*,*t*). The state of the system can be described by the distribution density *W*(*x*,*ϕ*,*t*) which is determined by the distribution density of the parameter *n*(*x*) and the conditional distribution density of oscillators *w*(*x*,*ϕ*,*t*), written as


According to the idea presented in [14], three new variables *ρ*(*x*,*t*), *Φ*(*x*,*t*), *Ψ*(*x*,*t*) and constants of motion *ψ*(*x*) are introduced to the original system of equations via the transformation
36

which transforms the time-dependent density *w*(*x*,*ϕ*,*t*) to a stationary density *σ*(*x*,*ψ*) with the new variables *Φ*(*x*,*t*), *Ψ*(*x*,*t*) and *ρ*(*x*,*t*) which satisfy the following
373839

As three new variables are added to the system, additional constraints have to be defined to guarantee that the transformation (36) determines *ρ*, *Φ*, *Ψ* uniquely. These constraints are defined in [14, 16] as follows:


In section “Mathematical model”, we apply this ansatz to populations of oscillators with complex mean field coupling. It is therefore important to determine the order parameters of each subpopulation. In [16] it was shown that


holds. This simplifies further if the constants of motion are distributed uniformly in which case *γ*(*x*)=1 (see [16]).

In [15, 25] the same general model (35) is treated with a different ansatz. Their idea was to extend the density function *W*(*x*,*ϕ*,*t*) to a Fourier series:


where *c*.*c*. denotes complex conjugate. Ott and Antonsen have shown that the continuity equation that expresses the conservation of the number of oscillators is fullfilled if the Fourier coefficients *f*_*m*_ can be written in terms of a single function *F*^*m*^. This set of solutions is the so-called OA-manifold. Pikovsky and Rosenblum argued in [16] that the OA-manifold corresponds to the case of uniformly distributed constants of motion in the Watanabe-Strogatz Theory. Ott and Antonsen discussed in [25] that the OA-manifold is the only attractive region in terms of long-time evolution under the assumption that the parameter distribution *n*(*x*) is continuous. This makes it possible to reduce the systems to the OA-manifold for purposes of a long-time analysis.

## References

[1] Liddle PF (1987). **The symptoms of chronic schizophrenia. A re-examination of the positive-negative dichotomy**. Br J Psychiatry.

[2] Green MF (1996). **What are the functional consequences of neurocognitive deficits in schizophrenia?**. Am J Psychiatry.

[3] Mueser KT, McGurk SR (2004). **Schizophrenia**. Lancet.

[4] Carlsson A (2006). **The neurochemical circuitry of schizophrenia**. Pharmacopsychiatry.

[5] an der Heiden U (2006). **Schizophrenia as a dynamical disease**. Pharmacopsychiatry.

[6] Mackey MC, an der Heiden U (1982). **Dynamical diseases and bifurcations: understanding functional disorder in physiological systems**. Funkt Biol Med.

[7] Zendehrouh S, Bakouie F, Gharibzadeh S, Rostami A (2010). **Mathematical modeling of schizophrenia**. J Paramedical Sci.

[8] Loh M, Rolls ET, Deco G (2007). **A dynamical systems hypothesis of schizophrenia**. PLoS Comput Biol.

[9] Rolls E, Loh M, Deco G, Winterer G (2008). **Computational models of schizophrenia and dopamine modulation in the prefrontal cortex**. Nat Rev Neurosci.

[10] Brodersen K, Deserno L, Schlagenhauf F, Penny W, Buhmann J, Stephan K (2014). **Dissecting psychiatric spectrum disorders by generative embedding**. NeuroImage: Clinical.

[11] Deserno L, Sterzer P, Wüstenberg T, Heinz A, Schlagenhauf F (2012). **Reduced prefrontal-parietal effective connectivity and working memory deficits in schizophrenia**. J Neurosci.

[12] Brockhaus-Dumke A, Müller R, Faigle U, Klosterkötter J (2008). **Sensory gating revisited: relation between brain oscillations and auditory evoked potentials in schizophrenia**. Schizophr Res.

[13] Popovych S, Küpper T, Müller R, Brockhaus-Dumke A (2009). **Modelling disturbance in early sensory processing in schizophrenia**. GAMM-Mitteilungen.

[14] Watanabe S, Strogatz S (1994). **Constants of motion for superconducting Josephson arrays**. Physica D.

[15] Ott E, Antonsen T (2009). **Long time evolution of phase oscillator systems**. Chaos.

[16] Pikovsky A, Rosenblum M (2011). **Dynamics of heterogeneous oscillator ensembles in terms of collective variables**. Physica D.

[17] Potter D, Summerfelt A, Gold J, Buchanan RW (2006). **Review of clinical correlates of P50 sensory gating abnormalities in patients with schizophrenia**. Schizophr Bull.

[18] Teplan M (2002). **Fundamentals of EEG measurement**. Meas Sci Rev.

[19] Tass PA (2002). **Stimulus-locked transient phase dynamics, synchronization and desynchronization of two oscillators**. Europhys Lett.

[20] Llinás R, Ribary U, Jeanmonod D, Kronberg E, Mitra P (1999). **Thalamocortical dyrhythmia: a neurological and neuropsychiatric syndrome characterized by magnetoencephalography**. PNAS.

[21] Ferrarelli F, Tononi G (2011). **The thalamic reticular nucleus and schizophrenia**. Schizophr Bull.

[22] McAlonan K, Brown VJ, Bowman EM (2000). **Thalamic reticular nucleus activation reflects attentional gating during classical conditioning**. J Neurosci.

[23] Krause M, Hoffmann WE, Hajós M (2003). **Auditory sensory gating in hippocampus and reticular thalamic neurons in anesthetized rats**. Biol Psychiatry.

[24] Pikovsky A, Rosenblum M (2008). **Partially integrable dynamics of hierarchical populations of coupled oscillators**. Phys Rev Lett.

[25] Ott E, Antonsen T (2008). **Low dimensional behavior of large systems of globally coupled oscillators**. Chaos.

[26] Kim DJ, Bolbecker AR, Howell J, Rass O, Sporns O, Hetrick WP, Breier A, O’Donnell BF (2013). **Disturbed resting state EEG synchronization in bipolar disorder: a graph-theoretic analysis**. NeuroImage: Clinical.

[27] Pfurtscheller G, Pichler-Zalaudek K, Ortmayr B, Diez J, Reisecker F (1998). **Postmovement beta synchronization in patients with Parkinson’s Disease**. J Clin Neurophysiol.

[28] Stephan KE, Friston KJ, Frith CD (2009). **Dysconnection in schizophrenia: from abnormal synaptic plasticity to failures of self-monitoring**. Schizophr Bull.

[29] Friston KJ, Frith CD, Fletcher P, Liddle PF, Frackowiak RS (1996). **Functional topography: multidimensional scaling and functional connectivity in the brain**. Cereb Cortex.

[30] Lawrie SM, Buechel C, Whalley HC, Frith CD, Friston KJ, Johnstone EC (2002). **Reduced frontotemporal functional connectivity in schizophrenia associated with auditory hallucinations**. Biol Psychiatry.

[31] Meyer-Lindenberg AS, Olsen RK, Kohn PD (2005). **Regionally specific disturbance of dorsolateral prefrontal hippocampal functional connectivity in schizophrenia**. Arch Gen Psychiatry.

[32] Friston KJ (1996). **Theoretical neurobiology and schizophrenia**. Br Med Bull.

[33] Friston KJ (1998). **The disconnection hypothesis**. Schizophr Res.

[34] Peet M, Brind J, Ramchand CN, Shah S, Vankar GK (2001). **Two double-blind placebo-controlled pilot studies of eicosapentaenoic acid in the treatment of schizophrenia**. Schizophr Res.

[35] Marx CE, Keefe RSE, Buchanan RW, Hamer RM, Kilts JD, Bradford DW, Strauss JL, Naylor JC, Payne VM, Lieberman JA, Savitz AJ, Leimone LA, Dunn L, Porcu P, Morrow AL, Shampine LJ (2009). **Proof-of-concept trial with the neurosteroid pregnenolone targeting cognitive and negative symptoms in schizophrenia**. Neuropsychopharmacology.

[36] Ritsner MS, Gibel A, Shleifer T, Boguslavsky I, Zayed A, Maayan R, Weizman A, Lerner V (2010). **Pregnenolone and dehydroepiandrosterone as an adjunctive treatment in schizophrenia and schizoaffective disorder: an 8-week, double-blind, randomized, controlled, 2-center, parallel-group trial**. J Clin Psychiatry.

[37] Carlsson A (1978). **Antipsychotic drugs, neurotransmitters, and schizophrenia**. Am J Psychiatry.

[38] Friston K, Harrison L, Penny W (2003). **Dynamic causal modelling**. NeuroImage.

[39] Komarov M, Pikovsky A (2011). **Effects of nonresonant interaction in ensembles of phase oscillators**. Phys Rev E.

[40] Komarov M, Pikovsky A (2003). **Dynamics of multifrequency oscillator communities**. Phys Rev Lett.

